# Severe Macrocytic Anemia Associated With a Novel *ALAS2* Mutation: A Case Report and Literature Review

**DOI:** 10.1002/ccr3.73529

**Published:** 2026-09-17

**Authors:** Haodong Cai, Kefeng Shen, Jin Wang, Jia Gu, Meilan Zhang, Min Xiao

**Affiliations:** ^1^ Department of Hematology, Tongji Hospital Tongji Medical College, Huazhong University of Science and Technology Wuhan China

**Keywords:** *ALAS2* mutation, AlphaFold 3, case report, macrocytic anemia, whole‐exome sequencing, X‐linked sideroblastic anemia

## Abstract

X‐linked sideroblastic anemia (XLSA), caused by pathogenic variants in *ALAS2*, typically presents as microcytic anemia in males. However, heterozygous females occasionally exhibit an atypical macrocytic phenotype, often leading to diagnostic ambiguity and confusion with nutritional anemias or myelodysplastic syndromes. This study aims to characterize a novel *ALAS2* variant and explore the possible mechanisms underlying macrocytosis in a female patient. We analyzed a 15‐year‐old female with an 8‐year history of refractory macrocytic anemia. Diagnostic evaluations included hematological profiling, iron metabolism studies, and bone marrow examination. Whole‐exome sequencing (WES) was employed to identify genetic variants, followed by Sanger validation. Protein structural modeling was performed using AlphaFold 3. The patient presented with severe macrocytic anemia, iron overload, and borderline folate status despite supplementation. WES identified a novel frameshift variant in *ALAS2*, c.1277dupT (p.M426Ifs*66), which supported the diagnosis of atypical XLSA. Structural modeling suggested that this variant alters the C‐terminal region and may affect the conformation of the catalytic site, including the spatial configuration around Lys391, potentially impairing enzymatic function. Additional variants in *DHX34*, *FANCI*, and *BRIP1* were also identified, although their potential contributions to the phenotype remain uncertain because functional evidence is lacking. In conclusion, we identified a novel, predicted loss‐of‐function variant in *ALAS2* associated with early‐onset macrocytic XLSA. Macrocytosis in this case may reflect a proposed “high‐turnover” failure involving intramedullary attrition of mutant clones and stress‐induced premature maturation of wild‐type clones; however, this mechanism remains speculative. Secondary nutrient depletion and additional genetic variants may also contribute, although their roles require further validation. This case underscores the value of genomic sequencing in resolving atypical, refractory anemias in female patients and preventing long‐term misdiagnosis.

## Key Clinical Message

Macrocytic anemia with borderline folate status may obscure XLSA in heterozygous females. In patients with persistent unexplained macrocytosis and iron overload, comprehensive genetic testing, including WES when initial evaluation is unrevealing, may facilitate diagnosis.

## 1 Introduction

X‐linked sideroblastic anemia (XLSA) is a rare genetic disorder, yet it remains the most prevalent form of hereditary sideroblastic anemia. XLSA is caused by pathogenic variants in the *ALAS2* gene, which impair heme biosynthesis within erythroid tissues [1, 2, 3]. This metabolic disruption leads to anemia and is characterized by the aberrant accumulation of iron within mitochondria, resulting in the formation of diagnostic ring sideroblasts in the bone marrow [4].

Owing to the X‐chromosomal localization of *ALAS2*, XLSA typically follows an X‐linked recessive inheritance pattern [5]. While hemizygous males are predominantly affected, heterozygous females often remain asymptomatic or exhibit only mild anemia. Consequently, male offspring in affected families are at a higher risk of phenotypic expression. Patients often present with symptoms of chronic anemia, such as fatigue, lethargy, and pallor [5]. Laboratory findings usually indicate microcytic hypochromic anemia, with iron metabolism studies showing elevated serum ferritin and bone marrow cytology revealing positive iron staining for ring sideroblasts [6].

Despite this typical presentation, several cases have reported female patients who develop the disease despite carrying only a monoallelic alteration [7, 8, 9, 10, 11]. Furthermore, a subset of female patients exhibits a macrocytic phenotype, which frequently precipitates diagnostic ambiguity and may lead to clinical misinterpretation prior to definitive genetic characterization [9, 10, 12]. We report the case of a young female patient whose persistent macrocytic anemia and concomitant folate deficiency were initially attributed to nutritional factors. Following the failure of empiric folate and vitamin B12 supplementation to elicit a hematological response, genomic investigation was performed. The identification of a novel *ALAS2* frameshift mutation supported the diagnosis of X‐linked sideroblastic anemia (XLSA). Notably, Whole Exome Sequencing (WES) further revealed multiple potentially pathogenic co‐occurring variants, although their relevance to the phenotype remains uncertain. By presenting this case, we aim to characterize a novel *ALAS2* frameshift mutation and leverage this clinical encounter, alongside a comprehensive literature review, to explore possible explanations underlying macrocytosis in XLSA.

## 2 Case History/Examination

A 15‐year‐old female presented to our hospital with an 8‐year history of persistent anemia. Her condition was first identified during a routine physical examination 8 years prior, at which time she was diagnosed with iron‐deficiency anemia. However, despite oral iron supplementation, her hemoglobin levels failed to improve. Two years ago, the patient began experiencing symptoms of dizziness and fatigue. Subsequent laboratory investigations revealed macrocytic anemia, and additional testing identified a concurrent folate deficiency. Bone marrow aspiration at that time showed mild dyserythropoiesis, leading to a diagnosis of nutritional anemia. She was initially prescribed oral folic acid monotherapy (5 mg three times daily), followed by combined treatment with folic acid (5 mg three times daily) and mecobalamin (0.5 mg three times daily). Because these treatments were administered before referral to our institution, the exact duration of each regimen and the patient's adherence could not be reliably verified from the available records.

Following a period of therapy, follow‐up examinations indicated that the anemia remained refractory, with hemoglobin (Hb) levels persisting at approximately 80 g/L. After an additional year of treatment, her Hb levels further declined to approximately 60 g/L, prompting her referral to our facility for further diagnostic evaluation.

Upon admission, laboratory investigations revealed a Hb level of 59 g/L. Erythrocyte indices were significant for macrocytosis, with a mean corpuscular volume (MCV) of 113.1 fL, mean corpuscular hemoglobin (MCH) of 36.9 pg., and mean corpuscular hemoglobin concentration (MCHC) of 326 g/L. The red blood cell distribution width‐coefficient of variation was 17.2%, and the red blood cell distribution width‐standard deviation was 70.2 fL.

Reticulocyte analysis showed a mildly elevated RET% (2.48%) with an absolute reticulocyte count of 0.040 × 10^12^/L. Using the concurrent hematocrit of 18.1% and a maturation correction factor of 2.5, the calculated reticulocyte production index (RPI) was approximately 0.40. This low RPI indicated an inadequate marrow response relative to the severity of anemia and was consistent with ineffective erythropoiesis. White blood cell and platelet counts were both within normal limits.

## 3 Methods

### 3.1 Investigations

Further investigations regarding the etiology of anemia were performed. Iron metabolism studies showed a serum ferritin level of 871.7 μg/L, serum iron of 44.12 μmol/L, and an unsaturated iron‐binding capacity of < 3.0 μmol/L, collectively reflecting appreciable iron storage elevation.

Nutritional markers revealed a serum folate level of 4.14 ng/mL (slightly above the lower limit of the reference range of 4.0 ng/mL) and a vitamin B12 level of 395 pg/mL. Given the patient's history of continuous supplementation, this borderline folate level was interpreted as a persistent state of functional folate deficiency.

Regarding markers of hemolysis, the plasma free hemoglobin was elevated at 47.40 mg/L (reference range: < 40.00 mg/L) and haptoglobin was markedly suppressed at < 0.06 g/L (reference range: 0.36–1.95 g/L), suggesting the presence of hemolytic activity. The erythrocyte osmotic fragility test did not reveal any significant increase in osmotic fragility. Furthermore, the Coombs test was negative, and the serum erythropoietin (EPO) was significantly elevated at 213.62 mIU/mL (reference range: 2.59–18.50 mIU/mL). The ceruloplasmin level was 0.205 g/L (reference range: 0.22–0.58 g/L).

Biochemical investigations revealed elevations in both indirect bilirubin (28.9 μmol/L; reference range: ≤ 12.9 μmol/L) and total bilirubin (37.7 μmol/L; reference range: ≤ 21 μmol/L). These findings further support the presence of hemolytic activity or ineffective erythropoiesis.

**FIGURE 1:**
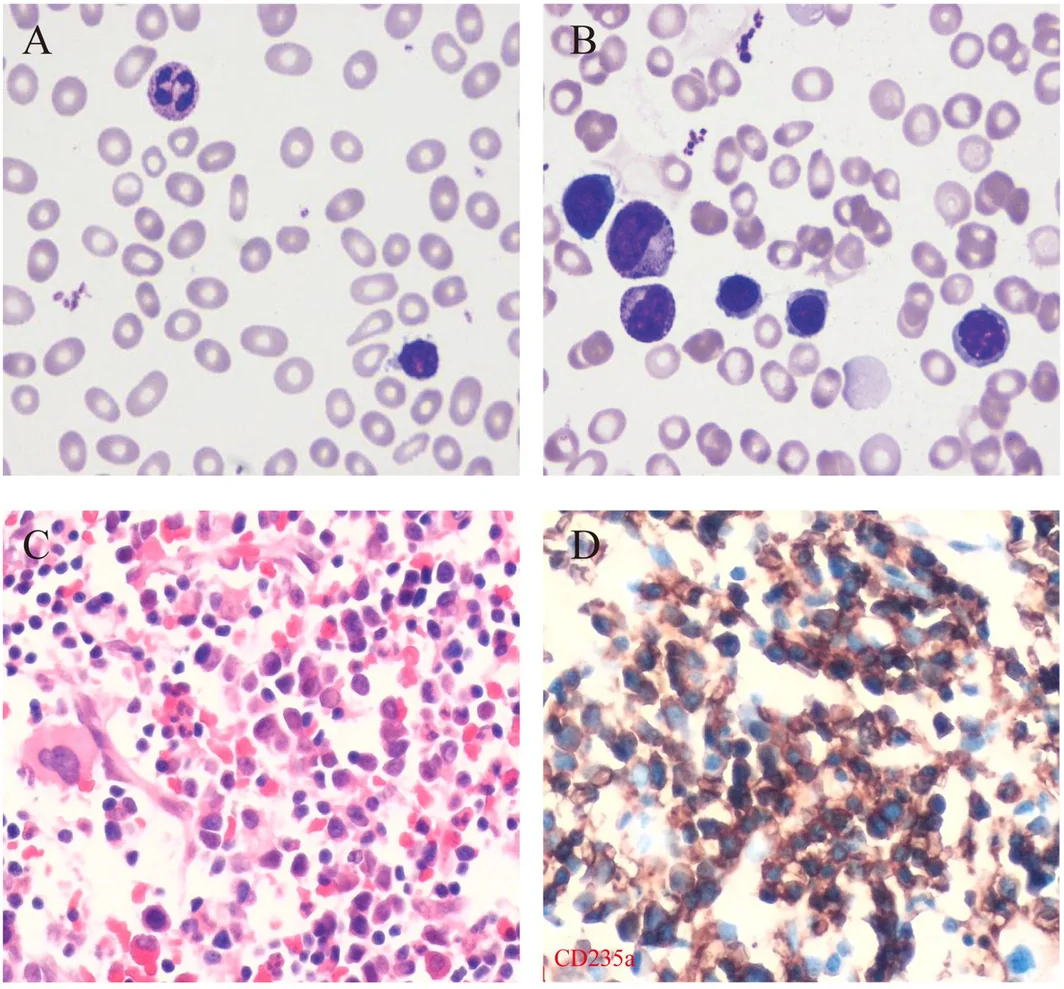
Morphological and immunohistochemical examination of peripheral blood and bone marrow. (A) Peripheral blood smear (Wright–Giemsa stain, ×1000) displays significant anisocytosis and poikilocytosis. Erythrocytes exhibit diverse morphologies, including ovalocytes and dacrocytes (teardrop cells); a subset of cells shows an enlarged central pallor. (B) Bone marrow aspirate (Wright–Giemsa stain, ×1000) reveals erythroid hyperplasia, predominantly composed of intermediate and late erythroblasts with unremarkable morphology. Mature erythrocytes exhibit an enlarged central pallor, consistent with peripheral findings. (C) Bone marrow biopsy (Hematoxylin and Eosin stain, ×400) demonstrates a hyperplastic erythroid lineage with a predominance of polychromatic and orthochromatic normoblasts. (D) Immunohistochemical staining shows strong diffuse positivity for CD235a within the erythroid series.

Morphological analysis of peripheral blood reveals erythrocytes of varying sizes and diverse shapes (Figure 1A). Bone marrow aspiration showed relative erythroid hyperplasia, predominantly involving intermediate and late erythroblasts (Figure 1B). Bone marrow biopsy was markedly hypercellular (> 90%), with erythroid predominance (Figure 1C,D). Importantly, no increase in myeloblasts or evidence of monoclonal lymphoid populations was identified via bone marrow cytology, histology, or flow cytometry.

**FIGURE 2:**
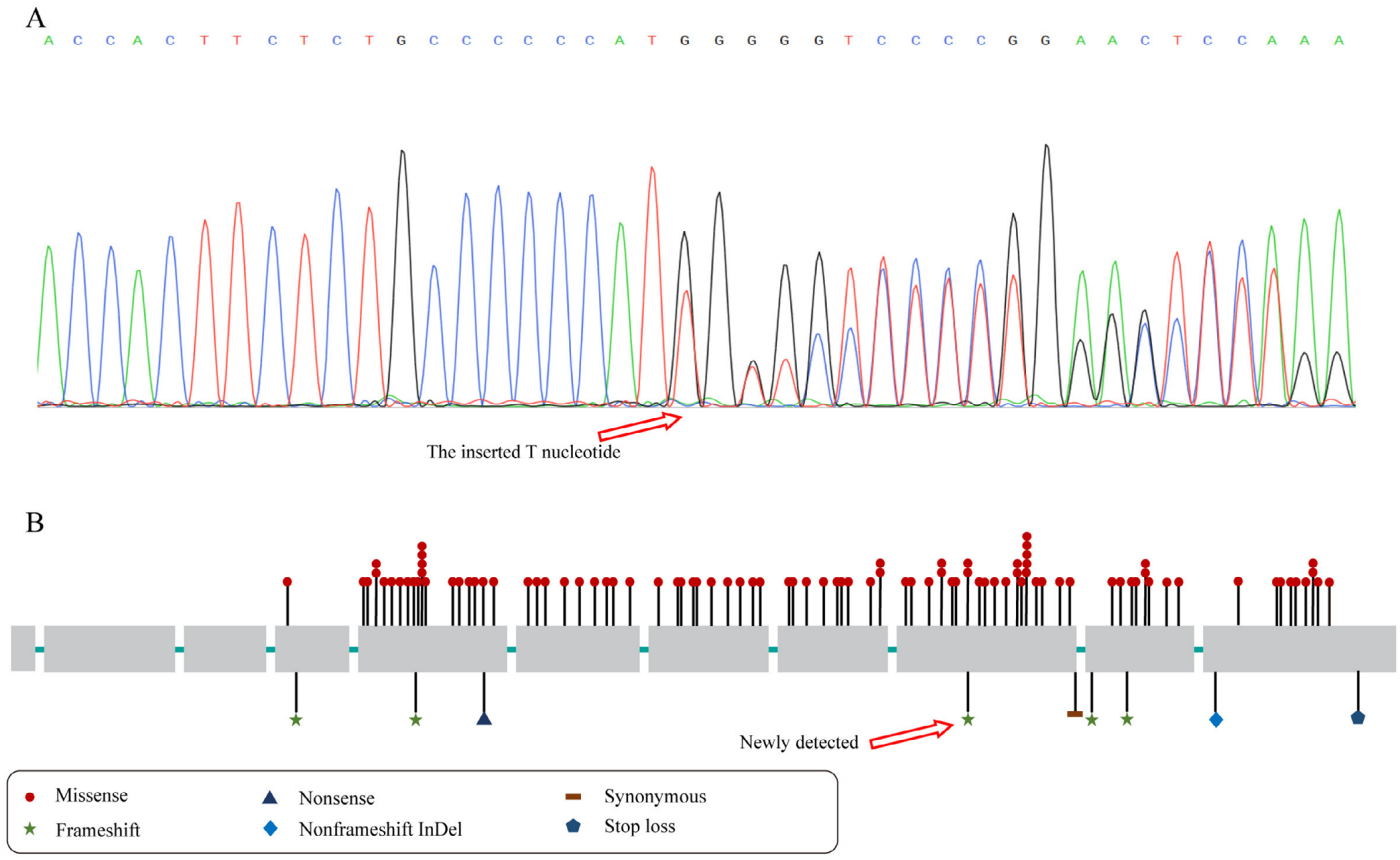
Mutational analysis of *ALAS2*. (A) Sanger sequencing chromatograms confirmed a thymine (T) insertion, resulting in a frameshift mutation. (B) Schematic representation of reported pathogenic mutations in the *ALAS2* coding sequence (CDS). Mutations labeled “Newly detected” indicate novel variants identified in the present study.

**FIGURE 3:**
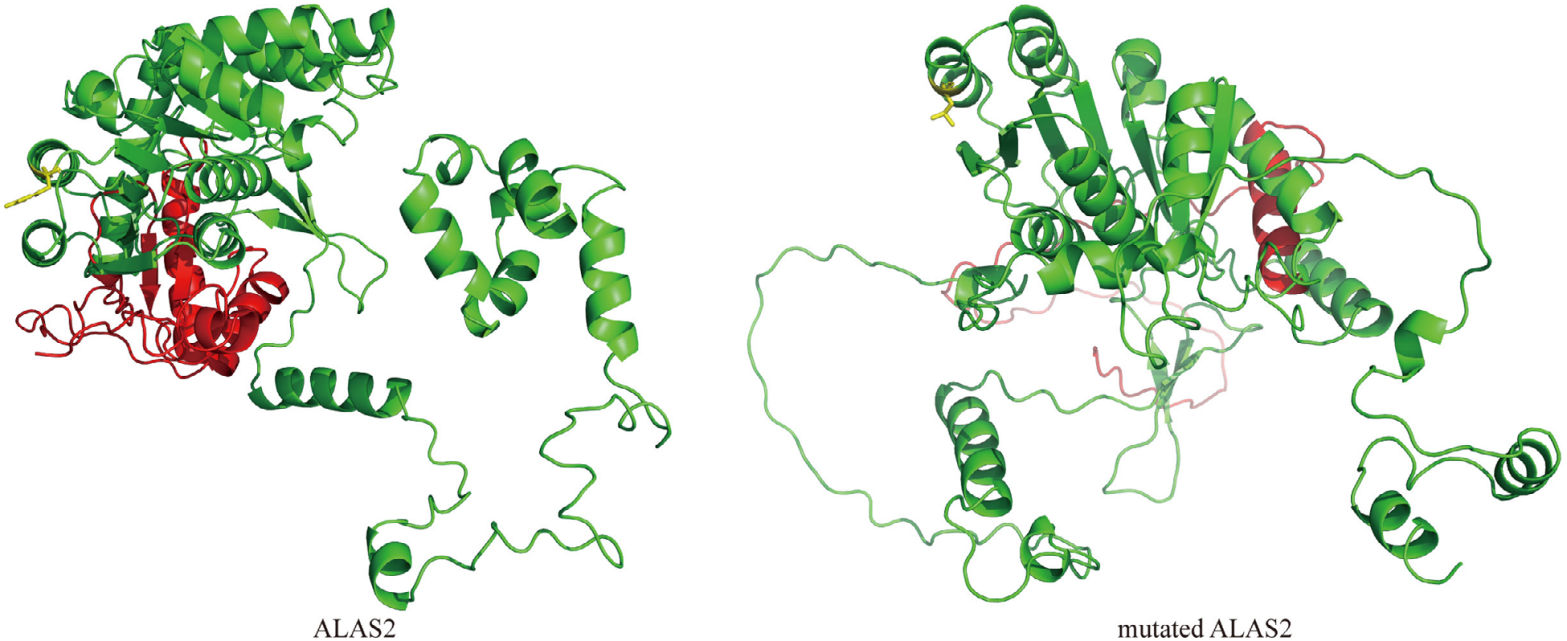
Structural modeling and comparison of wild‐type and mutant ALAS2 proteins. The left panel presents the wild‐type ALAS2 protein structure, while the right panel displays the predicted structure of the truncated mutant. The regions highlighted in red indicate the structural segments impacted by the mutation (pre‐mutation in the left panel vs. post‐mutation/truncation in the right panel). The residues highlighted in yellow represent Lys391, the catalytic lysine involved in pyridoxal 5′‐phosphate binding.

Globin gene sequencing revealed no pathogenic mutations within the coding regions of the *HBA1*, *HBA2*, or *HBB* genes. However, WES performed on bone marrow mononuclear cells identified a frameshift insertion mutation in the *ALAS2* gene: c.1277dupT (p.M426Ifs*66) (NCBI Reference Sequence: NM_000032.4) which was validated by Sanger sequencing (Figure 2A). This frameshift truncation results in the loss of critical C‐terminal functional domains of the ALAS2 protein. In silico analysis using AlphaFold 3 [13] suggests that this frameshift mutation disrupts the three‐dimensional conformation of the active site, specifically affecting the spatial orientation of the critical Lys391 residue (Figure 3). Given that Lys391 is essential for the formation of the Schiff base linkage with PLP, its structural destabilization likely renders the ALAS2 enzyme catalytically inert. Such structural disruption is expected to significantly impair the binding affinity for the pyridoxal 5′‐phosphate (PLP) cofactor, presumably leading to a complete loss of enzymatic function. Based on these findings, the variant is classified as highly pathogenic, providing molecular confirmation for the clinical diagnosis of XLSA.

Meanwhile, WES identified two missense mutations in the *DHX34* gene: c.1300C>T (p.R434W) and c.3361T>C (p.C1121R) (NCBI Reference Sequence: NM_014681.5). Both variants are rare in the general population and are predicted to be deleterious by SIFT and PolyPhen‐2 algorithms, potentially increasing the risk of familial MDS/AML. Additionally, variants were detected in *FANCI* [c.3179T>C (p.I1060T); NM_001113378.1] and *BRIP1* [c.2258A>G (p.D753G); NM_032043.2]. These are currently classified as variants of uncertain significance (VUS) in the ClinVar database, although SIFT and PolyPhen‐2 computational tools predict them to be deleterious or potentially damaging. In the absence of segregation and functional data, their clinical significance and relationship to the hematologic phenotype remain uncertain.

### 3.2 Differential Diagnosis

XLSA should be differentiated from several inherited and acquired disorders that may present with ineffective erythropoiesis, macrocytic anemia, and/or iron overload. When ring sideroblasts are detected, an acquired myelodysplastic neoplasm, particularly MDS with an *SF3B1* mutation, should be considered. Unlike inherited XLSA, MDS is a clonal disorder usually supported by persistent cytopenia(s), multilineage dysplasia, cytogenetic abnormalities, and/or somatic mutations. In this patient, childhood onset, isolated anemia with preserved leukocyte and platelet counts, absence of increased myeloblasts, and identification of the *ALAS2* frameshift variant favored XLSA. However, because bone marrow iron staining was unavailable, ring sideroblasts could not be assessed.

Congenital dyserythropoietic anemia (CDA) represents another important differential diagnosis because it can cause early‐onset ineffective erythropoiesis, macrocytosis, and secondary iron overload. CDA is typically associated with characteristic erythroblast abnormalities, including multinuclearity and internuclear chromatin bridges, and variants in genes such as *CDAN1*, *SEC23B*, *KIF23*, and *KLF1*. The available clinical, morphological, and sequencing findings did not strongly support CDA. Pearson syndrome, caused by mitochondrial DNA deletions, usually presents in infancy with macrocytic or sideroblastic anemia, vacuolated marrow precursors, pancreatic dysfunction, and other multisystem abnormalities; these features were not evident in this patient.

Other inherited marrow disorders considered included Diamond–Blackfan anemia, GATA1‐related erythroid disorders, and telomere biology disorders. These disorders more commonly present with infancy‐onset red‐cell aplasia and congenital anomalies, thrombocytopenia or megakaryocytic abnormalities, or progressive multilineage marrow failure and syndromic features, respectively, none of which were apparent in this case. Persistent anemia despite folate supplementation, together with iron overload and the *ALAS2* finding, therefore supported atypical XLSA, although direct functional evidence for the novel variant remains lacking.

### 3.3 Treatment

Following the molecular diagnosis of XLSA, the patient continued to receive red blood cell transfusions as clinically indicated for symptomatic anemia. Serum ferritin and other iron‐related parameters were monitored, but iron chelation therapy had not been initiated during the available observation period.

## 4 Conclusion and Results

At the last evaluation at our institution, the patient remained transfusion‐dependent, and serum ferritin levels remained elevated, consistent with ongoing iron overload. Iron chelation therapy had not been initiated. The patient subsequently returned home and did not attend further follow‐up at our institution. Consequently, information regarding her subsequent transfusion requirements, hematologic status, iron burden, treatment, and clinical outcomes was unavailable at the time of manuscript preparation.

## 5 Discussion

To date, over 100 pathogenic variants of the *ALAS2* gene have been reported [14, 15] (Figure 2B). These mutations are distributed across nearly every exon and several intronic regions. Missense mutations constitute the predominant mutational type, accounting for approximately 90% of cases; other variants, such as frameshift mutations resulting from insertions or deletions and nonsense mutations, occur less frequently [15]. Additionally, synonymous mutations affecting splicing have been reported [16], albeit rarely. The absence of distinct mutational “hotspots” within the *ALAS2* gene suggests that structural alterations at most sites are capable of compromising protein function (Figure 2B).

In this report, we present the case of a young, pediatric female patient successfully diagnosed with XLSA through genetic sequencing, which identified a mutation in the *ALAS2* gene. The identified variant, located in exon 9, involves a single‐nucleotide insertion (c.1277dupT), resulting in a profound frameshift and subsequent loss‐of‐function (LOF) of the *ALAS2* protein. Compared to the more prevalent missense mutations, this frameshift mutation exerts a more severe impact on protein integrity, likely leading to a complete loss of catalytic activity. This significant functional impairment potentially accounts for the uncommonly early clinical onset observed in this patient, which is notably earlier than that typically seen in female carriers [17].

As males possess only a single X chromosome, they lack allelic compensation, making them more susceptible to *ALAS2* mutations that lead to the overt clinical manifestations of XLSA. The resulting impairment of ALAS2 function disrupts heme biosynthesis, yielding a clinical profile characterized by microcytic hypochromic anemia, which closely mimics iron deficiency. Paradoxically, symptomatic cases in females with monoallelic *ALAS2* mutations are frequently reported as aforementioned. Investigations into X‐chromosome inactivation (XCI) patterns have revealed that these patients often exhibit skewed XCI, where the wild‐type *ALAS2* allele is predominantly inactivated, leaving the mutant allele to drive the phenotype [7, 17].

The typically later age of onset in female patients compared to males may suggest a dynamic clonal evolution. Specifically, erythroid progenitors carrying the non‐inactivated mutant *ALAS2* allele may possess a selective survival advantage, gradually outcompeting wild‐type cells over time and eventually leading to decompensation. Alternatively, this phenomenon may be attributed to stochastic clonal drift, wherein the subpopulation of cells with the active mutant allele randomly becomes dominant. The stochastic drift theory provides a plausible explanation for the incomplete penetrance observed among heterozygous female carriers.

Recent investigations into XCI patterns have yielded strikingly divergent findings. Contrary to established case reports, research suggests that X‐inactivation often selectively favors the mutant allele [14]. This revelation may fundamentally reshape our current understanding of XLSA pathogenesis in females. Since the wild‐type allele typically remains active, the manifestation of anemia in these patients likely involves alternative, occult mechanisms.

A distinctive clinical feature in a subset of female XLSA patients is the manifestation of macrocytic rather than microcytic anemia. While impaired hemoglobin synthesis is typically associated with compensatory hyperproliferation and microcytosis, macrocytosis suggests a reduction in the number of erythroid cell divisions. A previous study analyzing peripheral blood *ALAS2* mRNA sources has shown that transcripts are almost exclusively derived from the wild‐type (WT) allele [9]. This suggests that the ALAS2‐deficient clones are virtually incapable of undergoing terminal differentiation and maturation, and the circulating erythrocyte population is derived exclusively from WT lineages. Under the stress of hypoxia and elevated EPO levels, these WT‐derived cells might undergo premature maturation with fewer mitotic cycles, thereby generating a macrocytic phenotype.

However, hypoxia and increased EPO signaling may not fully account for the macrocytosis, because iron‐deficiency anemia and typical XLSA in males generally remain microcytic despite increased erythropoietic drive. This observation raises the possibility that additional, as yet undefined, factors may influence erythroblast proliferation or maturation. The patient also carried two *DHX34* variants and variants in *FANCI* and *BRIP1*. However, in the absence of functional evidence, their relevance to erythropoiesis and the macrocytic phenotype remains uncertain, and no causal or modifying role can currently be assigned to them. Further functional studies and additional well‐characterized cases are needed to determine whether genetic factors beyond *ALAS2* contribute to macrocytosis in heterozygous females with XLSA.

The documented folate deficiency may have contributed to the macrocytosis through impaired DNA synthesis. However, its relationship to the underlying erythroid disorder remains uncertain, and the lack of sustained hematologic improvement after folate supplementation suggests that folate deficiency alone did not account for the macrocytic phenotype.

Taken together, the available findings suggest that macrocytosis may reflect ineffective erythropoiesis involving intramedullary attrition of *ALAS2*‐deficient erythroblasts and fewer mitotic divisions among surviving wild‐type‐derived erythroblasts. However, this model remains hypothetical because X‐chromosome inactivation and functional studies were not performed. Clinically, XLSA should be considered in females with persistent macrocytic anemia, iron overload, and an inadequate reticulocyte response after common nutritional and acquired marrow disorders have been excluded.

Despite the insights gained, several limitations warrant consideration. Because the macrocytic phenotype was atypical for XLSA, iron staining was not performed as part of the initial bone marrow examination. After the genetic diagnosis, retrospective iron staining of the original bone marrow specimen was not undertaken because authorization for additional testing could not be obtained from the patient and her legal guardians. Consequently, the presence and proportion of ring sideroblasts could not be assessed, and classic XLSA morphology could not be confirmed. Iron staining during the initial evaluation might have prompted earlier consideration of XLSA. Second, although the patient and her legal guardians consented to publication of this case report, they declined familial genetic testing and did not authorize additional analyses of the available specimens, including X‐chromosome inactivation testing. Consequently, the inheritance pattern of the *ALAS2* variant, whether it arose de novo, and the patient's X‐chromosome inactivation status could not be determined.

Furthermore, owing to the severity of the anemia, the patient was promptly initiated on a regular blood transfusion regimen. Consequently, the clinical response to pyridoxine (Vitamin B6) supplementation was not evaluated. Based on the proposed pathogenic mechanism—wherein the frameshift mutation likely leads to a complete loss of ALAS2 function—it is theoretically improbable that pyridoxine would have conferred any therapeutic benefit. Lastly, whether therapies aimed at stimulating the compensatory *ALAS2 wild‐type* clones, such as EPO or luspatercept, could prove effective in this context remains to be elucidated through further clinical validation. However, their therapeutic effects could not be evaluated because the patient did not return to our institution for further follow‐up.

In conclusion, we identified a novel *ALAS2* mutation that causes a frameshift, likely resulting in a partial or near‐complete loss of protein function. This variant manifested as an early‐onset phenotype characterized by macrocytic anemia and progressively severe clinical symptoms. Notably, the patient presented with folate deficiency and hemolytic features that were refractory to folate supplementation. The eventual diagnosis via WES underscores the critical diagnostic utility of genomic profiling in resolving complex and atypical cases of refractory anemia.

## Author Contributions

**Haodong Cai:** writing – original draft. **Kefeng Shen:** data curation. **Jin Wang:** writing – review and editing. **Jia Gu:** writing – review and editing. **Meilan Zhang:** writing – review and editing. **Min Xiao:** supervision.

## Funding

This study was supported by the National Natural Science Foundation of China (No. 82270203 to Min Xiao and No. 82000189 to Haodong Cai).

## Ethics Statement

All procedures, including patient informed consent and experimental protocols, were conducted in accordance with the ethical standards of the Declaration of Helsinki and the institutional guidelines of Tongji Hospital. The study was approved by the Institutional Ethics Committee of Tongji Hospital.

## Consent

Written informed consent for the publication of the patient's anonymized clinical information and images was obtained from her legal guardians, and assent was also obtained from the patient.

## Conflicts of Interest

The authors declare no conflicts of interest.

## Data Availability

The datasets used and/or analyzed during the current study are available from the corresponding author on reasonable request.
